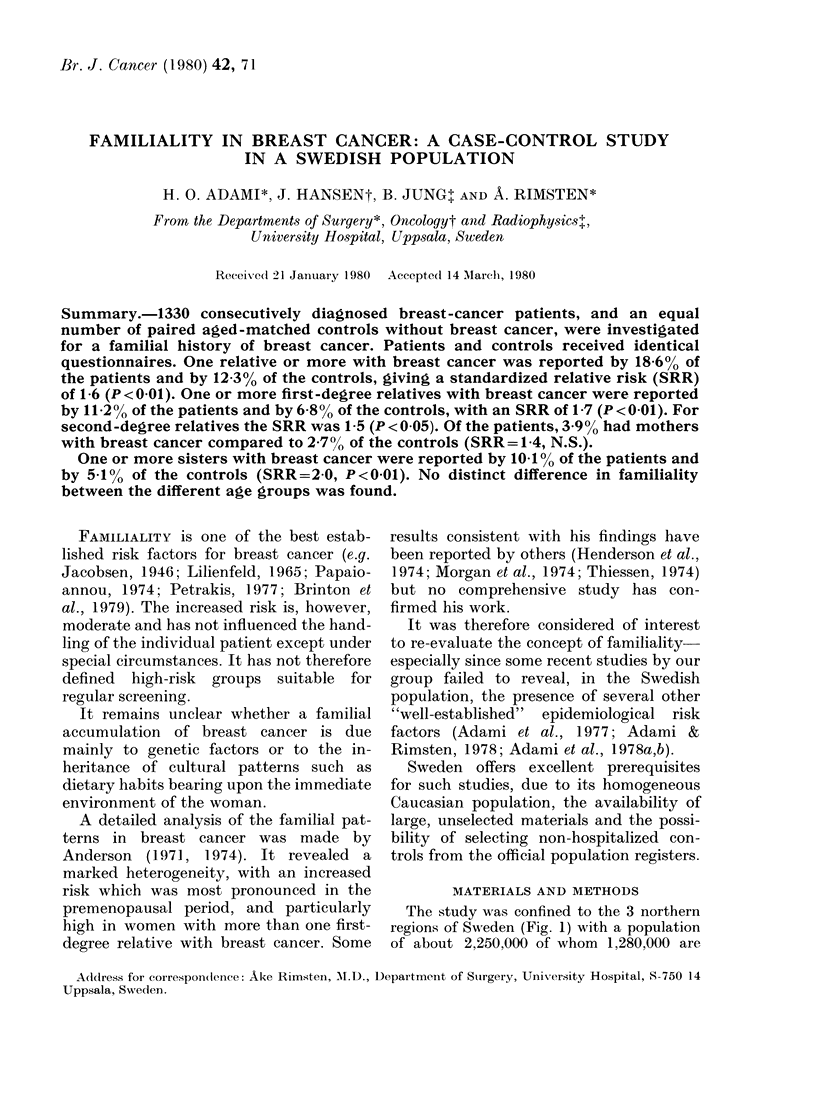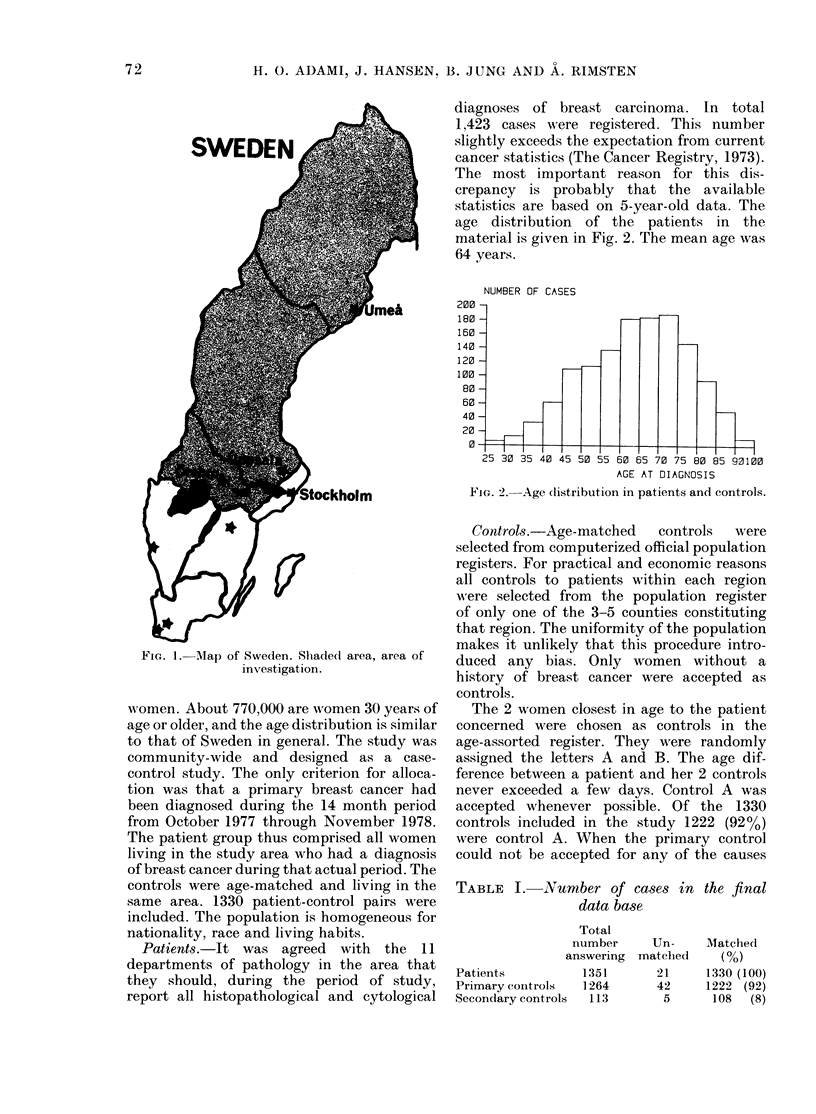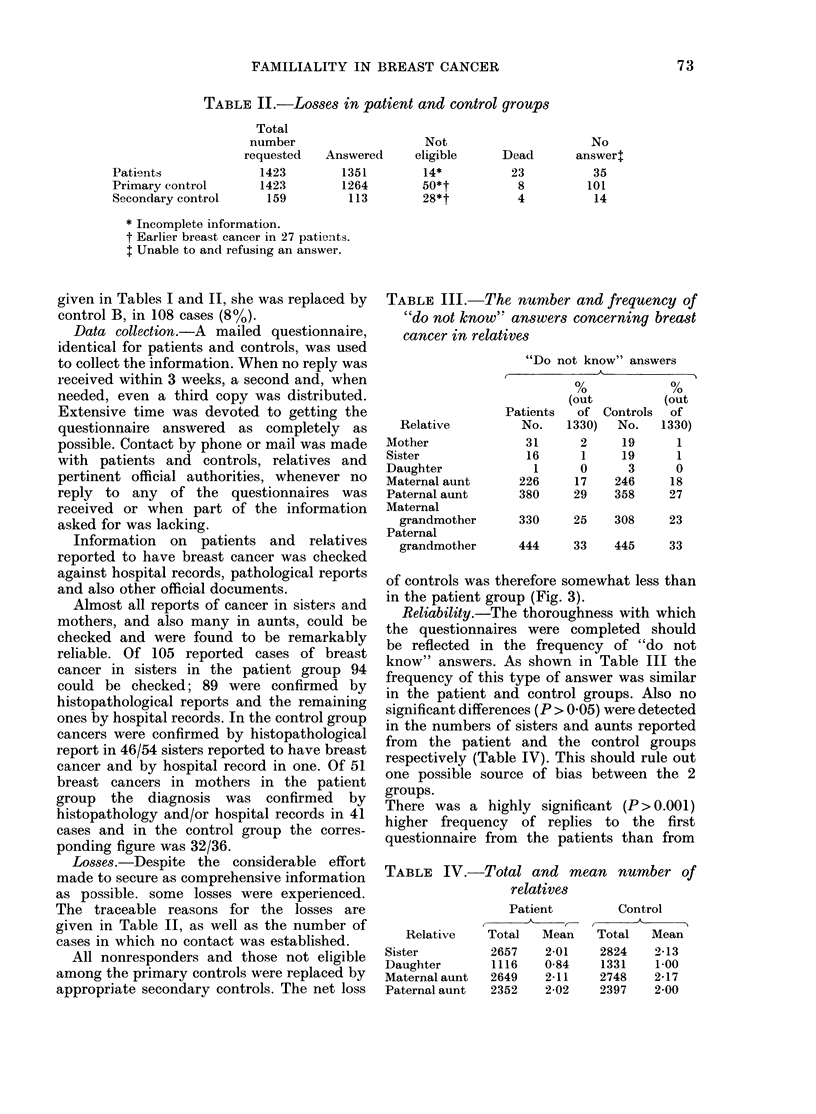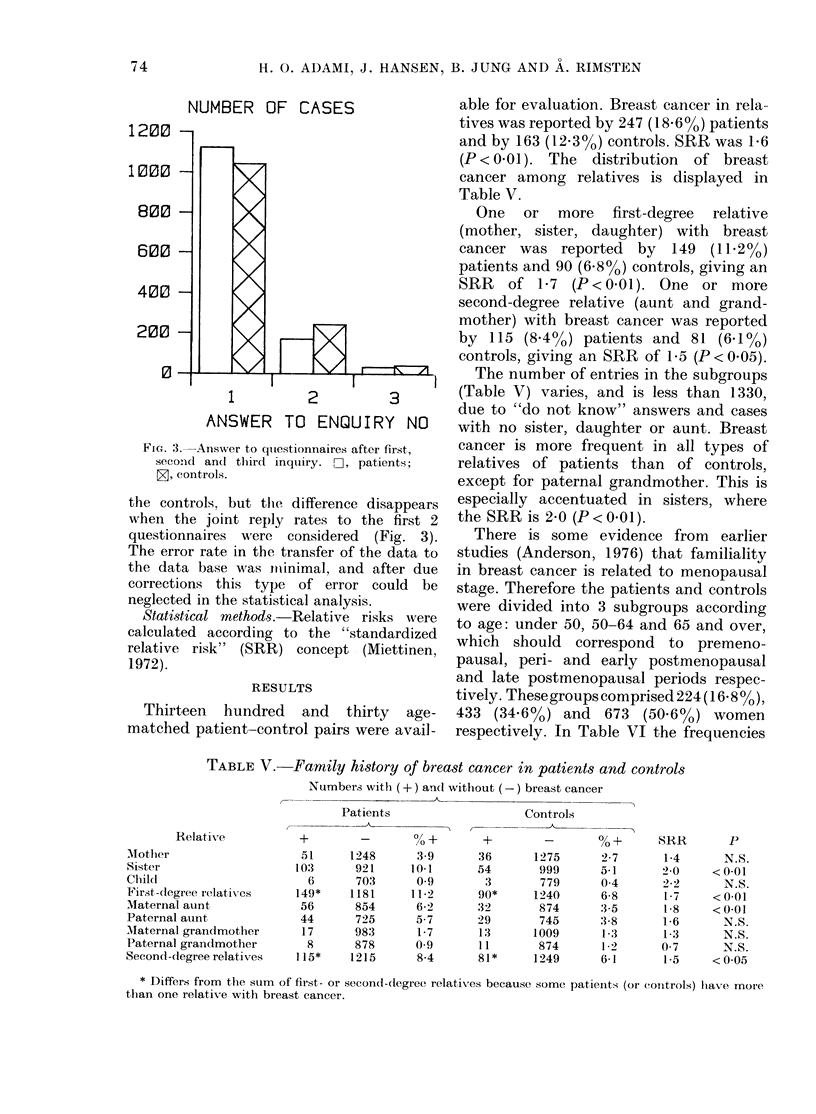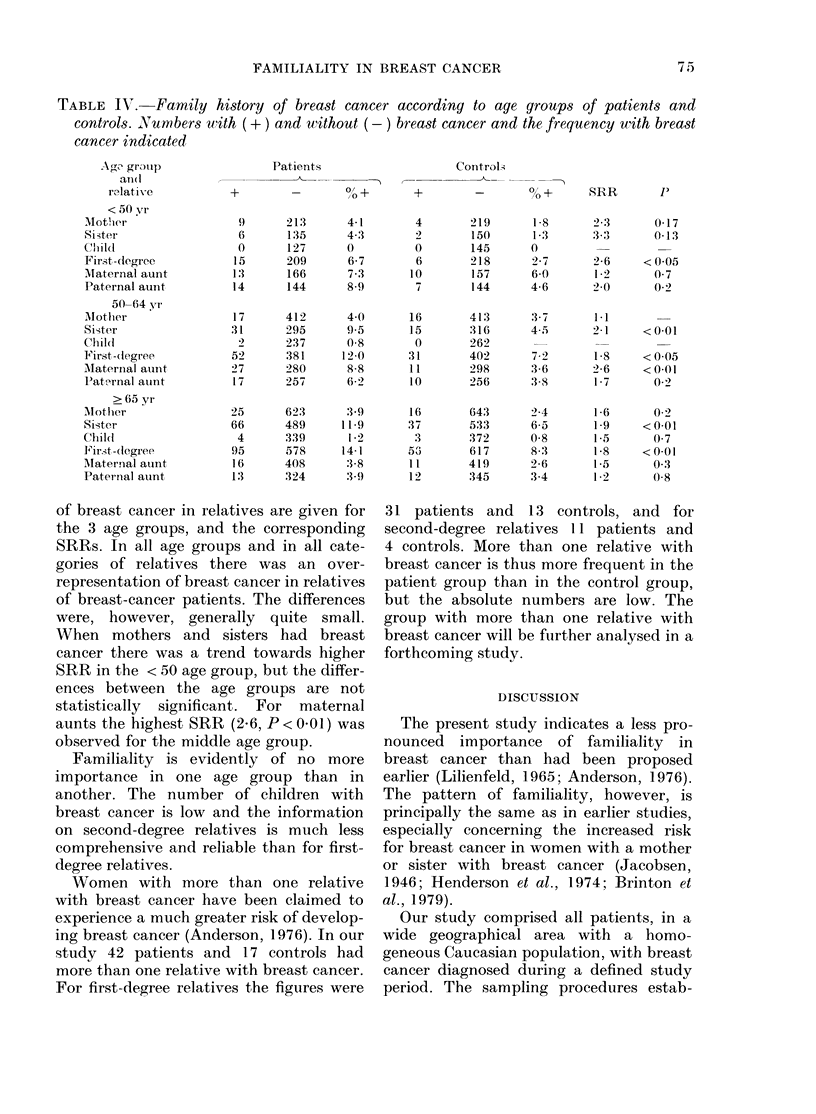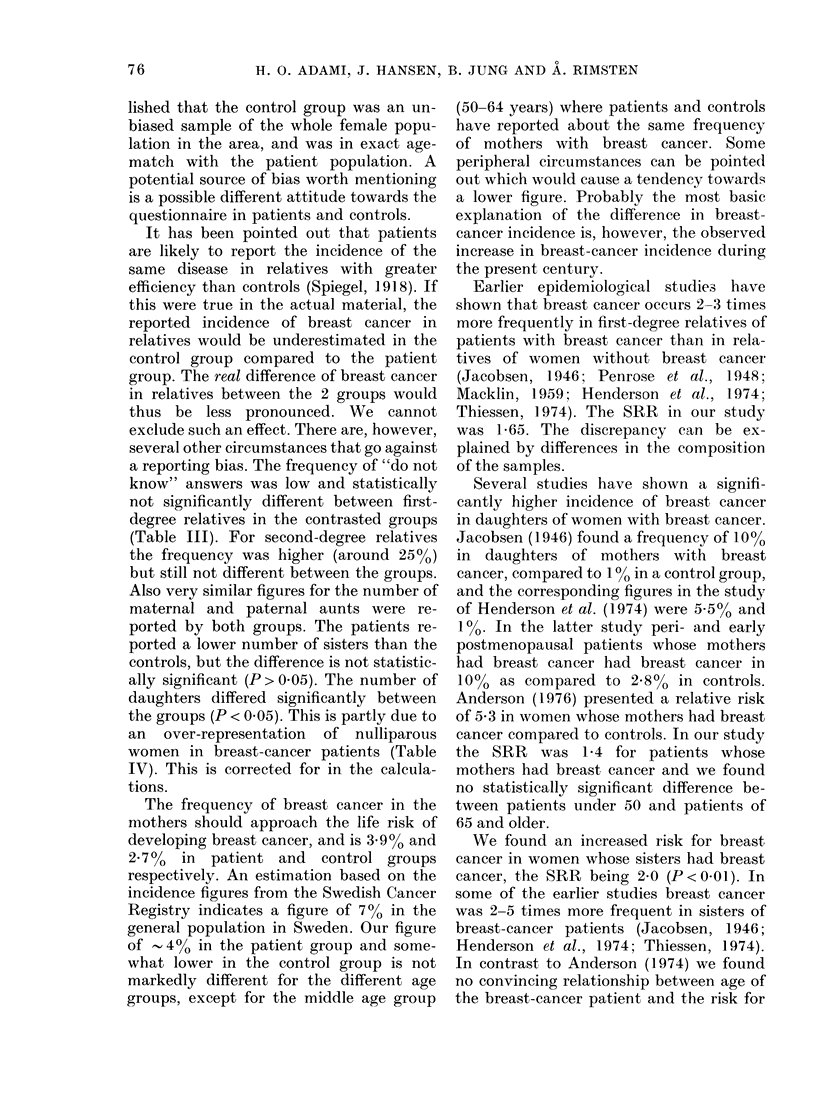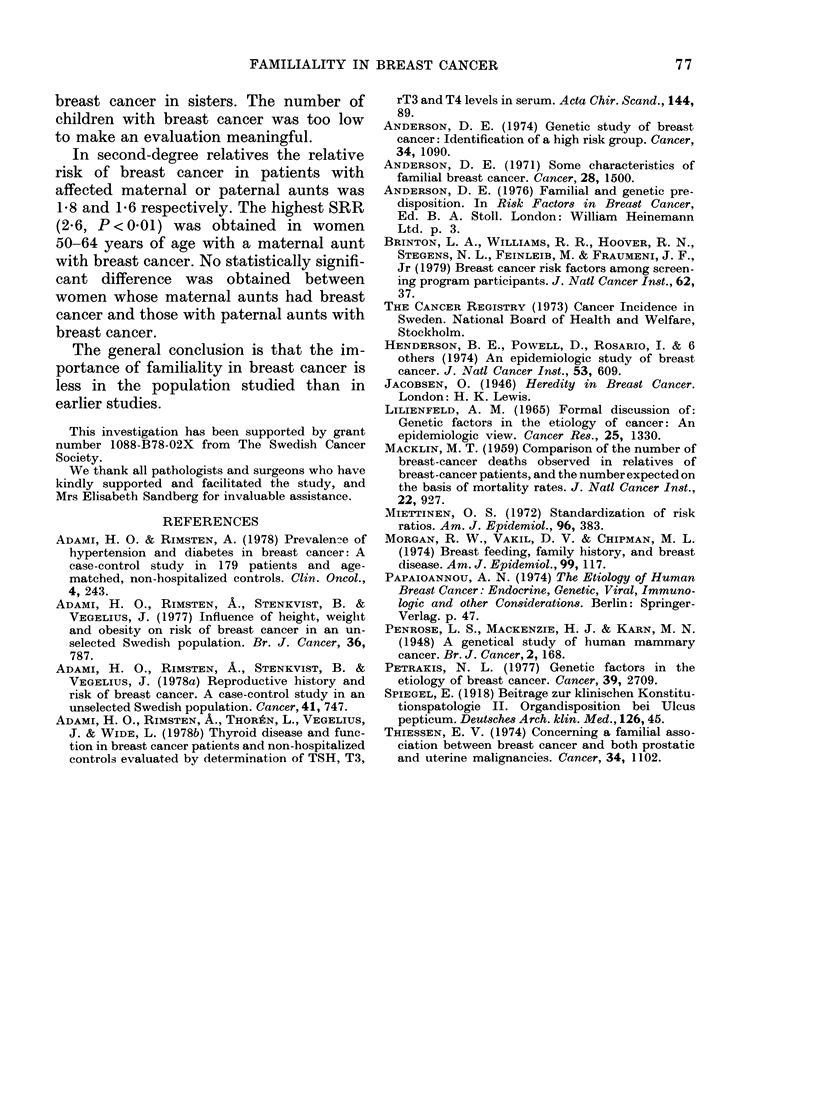# Familiality in breast cancer: a case-control study in a Sweden population.

**DOI:** 10.1038/bjc.1980.204

**Published:** 1980-07

**Authors:** H. O. Adami, J. Hansen, B. Jung, A. Rimsten

## Abstract

1330 consecutively diagnosed breast-cancer patients, and an equal number of paired aged-matched controls without breast cancer, were investigated for a familial history of breast cancer. Patients and controls received identical questionnaires. One relative or more with breast cancer was reported by 18.6% of the patients and by 12.3% of the controls, giving a standardized relative risk (SRR) of 1.6 (P < 0.01). One or more first-degree relatives with breast cancer were reported by 11.2% of the patients and by 6.8% of the controls, with an SRR of 1.7 (P < 0.01). For second-degree relatives the SRR was 1.5 (P < 0.05). Of the patients, 3.9% had mothers with breast cancer compared to 2.7% of the controls (SRR = 1.4, N.S.). One or more sisters with breast canceer were reported by 10.1% of the patients and by 5.1% of the controls (SRR = 2.0, P < 0.01). No distinct difference in familiality between the different age groups was found.


*Br. J. Cancer* (1980) **42**, 71

### FAMILIALITY IN BREAST CANCER: A CASE-CONTROL STUDY IN A SWEDISH POPULATION

H. O. ADAMI*, J. HANSEN†, B. JUNG‡ AND Å. RIMSTEN*

*From the Departments of Surgery*, Oncology† and Radiophysics‡, University Hospital, Uppsala, Sweden*

Received 21 January 1980 Accepted 14 March, 1980

**Summary.—1330 consecutively diagnosed breast-cancer patients, and an equal number of paired aged-matched controls without breast cancer, were investigated for a familial history of breast cancer. Patients and controls received identical questionnaires. One relative or more with breast cancer was reported by 18·6% of the patients and by 12·3% of the controls, giving a standardized relative risk (SRR) of 1·6 ($P<0·01$). One or more first-degree relatives with breast cancer were reported by 11·2% of the patients and by 6·8% of the controls, with an SRR of 1·7 ($P<0·01$). For second-degree relatives the SRR was 1·5 ($P<0·05$). Of the patients, 3·9% had mothers with breast cancer compared to 2·7% of the controls (SRR = 1·4, N.S.).**

**One or more sisters with breast cancer were reported by 10·1% of the patients and by 5·1% of the controls (SRR = 2·0, $P<0·01$). No distinct difference in familiality between the different age groups was found.**

FAMILIALITY is one of the best established risk factors for breast cancer (*e.g.* Jacobsen, 1946; Lilienfeld, 1965; Papaioannou, 1974; Petrakis, 1977; Brinton *et al.*, 1979). The increased risk is, however, moderate and has not influenced the handling of the individual patient except under special circumstances. It has not therefore defined high-risk groups suitable for regular screening.

It remains unclear whether a familial accumulation of breast cancer is due mainly to genetic factors or to the inheritance of cultural patterns such as dietary habits bearing upon the immediate environment of the woman.

A detailed analysis of the familial patterns in breast cancer was made by Anderson (1971, 1974). It revealed a marked heterogeneity, with an increased risk which was most pronounced in the premenopausal period, and particularly high in women with more than one first-degree relative with breast cancer. Some results consistent with his findings have been reported by others (Henderson *et al.*, 1974; Morgan *et al.*, 1974; Thiessen, 1974) but no comprehensive study has confirmed his work.

It was therefore considered of interest to re-evaluate the concept of familiality—especially since some recent studies by our group failed to reveal, in the Swedish population, the presence of several other "well-established" epidemiological risk factors (Adami *et al.*, 1977; Adami & Rimsten, 1978; Adami *et al.*, 1978*a,b*).

Sweden offers excellent prerequisites for such studies, due to its homogeneous Caucasian population, the availability of large, unselected materials and the possibility of selecting non-hospitalized controls from the official population registers.

### MATERIALS AND METHODS

The study was confined to the 3 northern regions of Sweden (Fig. 1) with a population of about 2,250,000 of whom 1,280,000 are

Address for correspondence: Åke Rimsten, M.D., Department of Surgery, University Hospital, S-750 14 Uppsala, Sweden.

FIG. 1.—Map of Sweden. Shaded area, area of investigation.

women. About 770,000 are women 30 years of age or older, and the age distribution is similar to that of Sweden in general. The study was community-wide and designed as a case-control study. The only criterion for allocation was that a primary breast cancer had been diagnosed during the 14 month period from October 1977 through November 1978. The patient group thus comprised all women living in the study area who had a diagnosis of breast cancer during that actual period. The controls were age-matched and living in the same area. 1330 patient-control pairs were included. The population is homogeneous for nationality, race and living habits.

*Patients.*—It was agreed with the 11 departments of pathology in the area that they should, during the period of study, report all histopathological and cytological diagnoses of breast carcinoma. In total 1,423 cases were registered. This number slightly exceeds the expectation from current cancer statistics (The Cancer Registry, 1973). The most important reason for this discrepancy is probably that the available statistics are based on 5-year-old data. The age distribution of the patients in the material is given in Fig. 2. The mean age was 64 years.

FIG. 2.—Age distribution in patients and controls.

*Controls.*—Age-matched controls were selected from computerized official population registers. For practical and economic reasons all controls to patients within each region were selected from the population register of only one of the 3–5 counties constituting that region. The uniformity of the population makes it unlikely that this procedure introduced any bias. Only women without a history of breast cancer were accepted as controls.

The 2 women closest in age to the patient concerned were chosen as controls in the age-assorted register. They were randomly assigned the letters A and B. The age difference between a patient and her 2 controls never exceeded a few days. Control A was accepted whenever possible. Of the 1330 controls included in the study 1222 (92%) were control A. When the primary control could not be accepted for any of the causes

TABLE I.—*Number of cases in the final data base*

| | Total number answering | Un-matched | Matched (%) |
|---|---|---|---|
| Patients | 1351 | 21 | 1330 (100) |
| Primary controls | 1264 | 42 | 1222 (92) |
| Secondary controls | 113 | 5 | 108 (8) |

TABLE II.—*Losses in patient and control groups*

| | Total number requested | Answered | Not eligible | Dead | No answer‡ |
|---|---|---|---|---|---|
| Patients | 1423 | 1351 | 14* | 23 | 35 |
| Primary control | 1423 | 1264 | 50*† | 8 | 101 |
| Secondary control | 159 | 113 | 28*† | 4 | 14 |

* Incomplete information.
† Earlier breast cancer in 27 patients.
‡ Unable to and refusing an answer.

given in Tables I and II, she was replaced by control B, in 108 cases (8%).

*Data collection.*—A mailed questionnaire, identical for patients and controls, was used to collect the information. When no reply was received within 3 weeks, a second and, when needed, even a third copy was distributed. Extensive time was devoted to getting the questionnaire answered as completely as possible. Contact by phone or mail was made with patients and controls, relatives and pertinent official authorities, whenever no reply to any of the questionnaires was received or when part of the information asked for was lacking.

Information on patients and relatives reported to have breast cancer was checked against hospital records, pathological reports and also other official documents.

Almost all reports of cancer in sisters and mothers, and also many in aunts, could be checked and were found to be remarkably reliable. Of 105 reported cases of breast cancer in sisters in the patient group 94 could be checked; 89 were confirmed by histopathological reports and the remaining ones by hospital records. In the control group cancers were confirmed by histopathological report in 46/54 sisters reported to have breast cancer and by hospital record in one. Of 51 breast cancers in mothers in the patient group the diagnosis was confirmed by histopathology and/or hospital records in 41 cases and in the control group the corresponding figure was 32/36.

*Losses.*—Despite the considerable effort made to secure as comprehensive information as possible. some losses were experienced. The traceable reasons for the losses are given in Table II, as well as the number of cases in which no contact was established.

All nonresponders and those not eligible among the primary controls were replaced by appropriate secondary controls. The net loss of controls was therefore somewhat less than in the patient group (Fig. 3).

TABLE III.—*The number and frequency of "do not know" answers concerning breast cancer in relatives*

| | "Do not know" answers | | | |
|---|---|---|---|---|
| Relative | Patients No. | % (out of 1330) | Controls No. | % (out of 1330) |
| Mother | 31 | 2 | 19 | 1 |
| Sister | 16 | 1 | 19 | 1 |
| Daughter | 1 | 0 | 3 | 0 |
| Maternal aunt | 226 | 17 | 246 | 18 |
| Paternal aunt | 380 | 29 | 358 | 27 |
| Maternal grandmother | 330 | 25 | 308 | 23 |
| Paternal grandmother | 444 | 33 | 445 | 33 |

*Reliability.*—The thoroughness with which the questionnaires were completed should be reflected in the frequency of "do not know" answers. As shown in Table III the frequency of this type of answer was similar in the patient and control groups. Also no significant differences ($P > 0{\cdot}05$) were detected in the numbers of sisters and aunts reported from the patient and the control groups respectively (Table IV). This should rule out one possible source of bias between the 2 groups.

There was a highly significant ($P > 0.001$) higher frequency of replies to the first questionnaire from the patients than from

TABLE IV.—*Total and mean number of relatives*

| | Patient | | Control | |
|---|---|---|---|---|
| Relative | Total | Mean | Total | Mean |
| Sister | 2657 | 2·01 | 2824 | 2·13 |
| Daughter | 1116 | 0·84 | 1331 | 1·00 |
| Maternal aunt | 2649 | 2·11 | 2748 | 2·17 |
| Paternal aunt | 2352 | 2·02 | 2397 | 2·00 |

FIG. 3.—Answer to questionnaires after first, second and third inquiry. □, patients; ⊠, controls.

the controls, but the difference disappears when the joint reply rates to the first 2 questionnaires were considered (Fig. 3). The error rate in the transfer of the data to the data base was minimal, and after due corrections this type of error could be neglected in the statistical analysis.

*Statistical methods.*—Relative risks were calculated according to the "standardized relative risk" (SRR) concept (Miettinen, 1972).

### RESULTS

Thirteen hundred and thirty age-matched patient–control pairs were available for evaluation. Breast cancer in relatives was reported by 247 (18·6%) patients and by 163 (12·3%) controls. SRR was 1·6 ($P < 0·01$). The distribution of breast cancer among relatives is displayed in Table V.

One or more first-degree relative (mother, sister, daughter) with breast cancer was reported by 149 (11·2%) patients and 90 (6·8%) controls, giving an SRR of 1·7 ($P < 0·01$). One or more second-degree relative (aunt and grandmother) with breast cancer was reported by 115 (8·4%) patients and 81 (6·1%) controls, giving an SRR of 1·5 ($P < 0·05$).

The number of entries in the subgroups (Table V) varies, and is less than 1330, due to "do not know" answers and cases with no sister, daughter or aunt. Breast cancer is more frequent in all types of relatives of patients than of controls, except for paternal grandmother. This is especially accentuated in sisters, where the SRR is 2·0 ($P < 0·01$).

There is some evidence from earlier studies (Anderson, 1976) that familiality in breast cancer is related to menopausal stage. Therefore the patients and controls were divided into 3 subgroups according to age: under 50, 50–64 and 65 and over, which should correspond to premenopausal, peri- and early postmenopausal and late postmenopausal periods respectively. These groups comprised 224 (16·8%), 433 (34·6%) and 673 (50·6%) women respectively. In Table VI the frequencies

TABLE V.—*Family history of breast cancer in patients and controls*

| | Numbers with (+) and without (−) breast cancer | | | | | | | |
|---|---|---|---|---|---|---|---|---|
| | Patients | | | Controls | | | | |
| Relative | + | − | %+ | + | − | %+ | SRR | *P* |
| Mother | 51 | 1248 | 3·9 | 36 | 1275 | 2·7 | 1·4 | N.S. |
| Sister | 103 | 921 | 10·1 | 54 | 999 | 5·1 | 2·0 | < 0·01 |
| Child | 6 | 703 | 0·9 | 3 | 779 | 0·4 | 2·2 | N.S. |
| First-degree relatives | 149* | 1181 | 11·2 | 90* | 1240 | 6·8 | 1·7 | < 0·01 |
| Maternal aunt | 56 | 854 | 6·2 | 32 | 874 | 3·5 | 1·8 | < 0·01 |
| Paternal aunt | 44 | 725 | 5·7 | 29 | 745 | 3·8 | 1·6 | N.S. |
| Maternal grandmother | 17 | 983 | 1·7 | 13 | 1009 | 1·3 | 1·3 | N.S. |
| Paternal grandmother | 8 | 878 | 0·9 | 11 | 874 | 1·2 | 0·7 | N.S. |
| Second-degree relatives | 115* | 1215 | 8·4 | 81* | 1249 | 6·1 | 1·5 | < 0·05 |

* Differs from the sum of first- or second-degree relatives because some patients (or controls) have more than one relative with breast cancer.

TABLE IV.—*Family history of breast cancer according to age groups of patients and controls. Numbers with (+) and without (−) breast cancer and the frequency with breast cancer indicated*

| Age group and relative | Patients | | | Controls | | | | |
|---|---|---|---|---|---|---|---|---|
| | + | − | %+ | + | − | %+ | SRR | $P$ |
| < 50 yr | | | | | | | | |
| Mother | 9 | 213 | 4·1 | 4 | 219 | 1·8 | 2·3 | 0·17 |
| Sister | 6 | 135 | 4·3 | 2 | 150 | 1·3 | 3·3 | 0·13 |
| Child | 0 | 127 | 0 | 0 | 145 | 0 | — | — |
| First-degree | 15 | 209 | 6·7 | 6 | 218 | 2·7 | 2·6 | < 0·05 |
| Maternal aunt | 13 | 166 | 7·3 | 10 | 157 | 6·0 | 1·2 | 0·7 |
| Paternal aunt | 14 | 144 | 8·9 | 7 | 144 | 4·6 | 2·0 | 0·2 |
| 50–64 yr | | | | | | | | |
| Mother | 17 | 412 | 4·0 | 16 | 413 | 3·7 | 1·1 | — |
| Sister | 31 | 295 | 9·5 | 15 | 316 | 4·5 | 2·1 | < 0·01 |
| Child | 2 | 237 | 0·8 | 0 | 262 | — | — | — |
| First-degree | 52 | 381 | 12·0 | 31 | 402 | 7·2 | 1·8 | < 0·05 |
| Maternal aunt | 27 | 280 | 8·8 | 11 | 298 | 3·6 | 2·6 | < 0·01 |
| Paternal aunt | 17 | 257 | 6·2 | 10 | 256 | 3·8 | 1·7 | 0·2 |
| ≥ 65 yr | | | | | | | | |
| Mother | 25 | 623 | 3·9 | 16 | 643 | 2·4 | 1·6 | 0·2 |
| Sister | 66 | 489 | 11·9 | 37 | 533 | 6·5 | 1·9 | < 0·01 |
| Child | 4 | 339 | 1·2 | 3 | 372 | 0·8 | 1·5 | 0·7 |
| First-degree | 95 | 578 | 14·1 | 56 | 617 | 8·3 | 1·8 | < 0·01 |
| Maternal aunt | 16 | 408 | 3·8 | 11 | 419 | 2·6 | 1·5 | 0·3 |
| Paternal aunt | 13 | 324 | 3·9 | 12 | 345 | 3·4 | 1·2 | 0·8 |

of breast cancer in relatives are given for the 3 age groups, and the corresponding SRRs. In all age groups and in all categories of relatives there was an over-representation of breast cancer in relatives of breast-cancer patients. The differences were, however, generally quite small. When mothers and sisters had breast cancer there was a trend towards higher SRR in the < 50 age group, but the differences between the age groups are not statistically significant. For maternal aunts the highest SRR (2·6, $P < 0{\cdot}01$) was observed for the middle age group.

Familiality is evidently of no more importance in one age group than in another. The number of children with breast cancer is low and the information on second-degree relatives is much less comprehensive and reliable than for first-degree relatives.

Women with more than one relative with breast cancer have been claimed to experience a much greater risk of developing breast cancer (Anderson, 1976). In our study 42 patients and 17 controls had more than one relative with breast cancer. For first-degree relatives the figures were 31 patients and 13 controls, and for second-degree relatives 11 patients and 4 controls. More than one relative with breast cancer is thus more frequent in the patient group than in the control group, but the absolute numbers are low. The group with more than one relative with breast cancer will be further analysed in a forthcoming study.

### DISCUSSION

The present study indicates a less pronounced importance of familiality in breast cancer than had been proposed earlier (Lilienfeld, 1965; Anderson, 1976). The pattern of familiality, however, is principally the same as in earlier studies, especially concerning the increased risk for breast cancer in women with a mother or sister with breast cancer (Jacobsen, 1946; Henderson *et al.*, 1974; Brinton *et al.*, 1979).

Our study comprised all patients, in a wide geographical area with a homogeneous Caucasian population, with breast cancer diagnosed during a defined study period. The sampling procedures established that the control group was an unbiased sample of the whole female population in the area, and was in exact age-match with the patient population. A potential source of bias worth mentioning is a possible different attitude towards the questionnaire in patients and controls.

It has been pointed out that patients are likely to report the incidence of the same disease in relatives with greater efficiency than controls (Spiegel, 1918). If this were true in the actual material, the reported incidence of breast cancer in relatives would be underestimated in the control group compared to the patient group. The *real* difference of breast cancer in relatives between the 2 groups would thus be less pronounced. We cannot exclude such an effect. There are, however, several other circumstances that go against a reporting bias. The frequency of "do not know" answers was low and statistically not significantly different between first-degree relatives in the contrasted groups (Table III). For second-degree relatives the frequency was higher (around 25%) but still not different between the groups. Also very similar figures for the number of maternal and paternal aunts were reported by both groups. The patients reported a lower number of sisters than the controls, but the difference is not statistically significant ($P > 0{\cdot}05$). The number of daughters differed significantly between the groups ($P < 0{\cdot}05$). This is partly due to an over-representation of nulliparous women in breast-cancer patients (Table IV). This is corrected for in the calculations.

The frequency of breast cancer in the mothers should approach the life risk of developing breast cancer, and is 3·9% and 2·7% in patient and control groups respectively. An estimation based on the incidence figures from the Swedish Cancer Registry indicates a figure of 7% in the general population in Sweden. Our figure of ~4% in the patient group and somewhat lower in the control group is not markedly different for the different age groups, except for the middle age group (50–64 years) where patients and controls have reported about the same frequency of mothers with breast cancer. Some peripheral circumstances can be pointed out which would cause a tendency towards a lower figure. Probably the most basic explanation of the difference in breast-cancer incidence is, however, the observed increase in breast-cancer incidence during the present century.

Earlier epidemiological studies have shown that breast cancer occurs 2–3 times more frequently in first-degree relatives of patients with breast cancer than in relatives of women without breast cancer (Jacobsen, 1946; Penrose *et al.*, 1948; Macklin, 1959; Henderson *et al.*, 1974; Thiessen, 1974). The SRR in our study was 1·65. The discrepancy can be explained by differences in the composition of the samples.

Several studies have shown a significantly higher incidence of breast cancer in daughters of women with breast cancer. Jacobsen (1946) found a frequency of 10% in daughters of mothers with breast cancer, compared to 1% in a control group, and the corresponding figures in the study of Henderson *et al.* (1974) were 5·5% and 1%. In the latter study peri- and early postmenopausal patients whose mothers had breast cancer had breast cancer in 10% as compared to 2·8% in controls. Anderson (1976) presented a relative risk of 5·3 in women whose mothers had breast cancer compared to controls. In our study the SRR was 1·4 for patients whose mothers had breast cancer and we found no statistically significant difference between patients under 50 and patients of 65 and older.

We found an increased risk for breast cancer in women whose sisters had breast cancer, the SRR being 2·0 ($P < 0{\cdot}01$). In some of the earlier studies breast cancer was 2–5 times more frequent in sisters of breast-cancer patients (Jacobsen, 1946; Henderson *et al.*, 1974; Thiessen, 1974). In contrast to Anderson (1974) we found no convincing relationship between age of the breast-cancer patient and the risk for breast cancer in sisters. The number of children with breast cancer was too low to make an evaluation meaningful.

In second-degree relatives the relative risk of breast cancer in patients with affected maternal or paternal aunts was 1·8 and 1·6 respectively. The highest SRR (2·6, $P < 0{\cdot}01$) was obtained in women 50–64 years of age with a maternal aunt with breast cancer. No statistically significant difference was obtained between women whose maternal aunts had breast cancer and those with paternal aunts with breast cancer.

The general conclusion is that the importance of familiality in breast cancer is less in the population studied than in earlier studies.

This investigation has been supported by grant number 1088-B78-02X from The Swedish Cancer Society.

We thank all pathologists and surgeons who have kindly supported and facilitated the study, and Mrs Elisabeth Sandberg for invaluable assistance.

### REFERENCES

Adami, H. O. & Rimsten, A. (1978) Prevalence of hypertension and diabetes in breast cancer: A case-control study in 179 patients and age-matched, non-hospitalized controls. *Clin. Oncol.*, **4**, 243.

Adami, H. O., Rimsten, Å., Stenkvist, B. & Vegelius, J. (1977) Influence of height, weight and obesity on risk of breast cancer in an unselected Swedish population. *Br. J. Cancer*, **36**, 787.

Adami, H. O., Rimsten, Å., Stenkvist, B. & Vegelius, J. (1978*a*) Reproductive history and risk of breast cancer. A case-control study in an unselected Swedish population. *Cancer*, **41**, 747.

Adami, H. O., Rimsten, Å., Thorén, L., Vegelius, J. & Wide, L. (1978*b*) Thyroid disease and function in breast cancer patients and non-hospitalized controls evaluated by determination of TSH, T3, rT3 and T4 levels in serum. *Acta Chir. Scand.*, **144**, 89.

Anderson, D. E. (1974) Genetic study of breast cancer: Identification of a high risk group. *Cancer*, **34**, 1090.

Anderson, D. E. (1971) Some characteristics of familial breast cancer. *Cancer*, **28**, 1500.

Anderson, D. E. (1976) Familial and genetic predisposition. In *Risk Factors in Breast Cancer*, Ed. B. A. Stoll. London: William Heinemann Ltd. p. 3.

Brinton, L. A., Williams, R. R., Hoover, R. N., Stegens, N. L., Feinleib, M. & Fraumeni, J. F., Jr (1979) Breast cancer risk factors among screening program participants. *J. Natl Cancer Inst.*, **62**, 37.

The Cancer Registry (1973) Cancer Incidence in Sweden. National Board of Health and Welfare, Stockholm.

Henderson, B. E., Powell, D., Rosario, I. & 6 others (1974) An epidemiologic study of breast cancer. *J. Natl Cancer Inst.*, **53**, 609.

Jacobsen, O. (1946) *Heredity in Breast Cancer.* London: H. K. Lewis.

Lilienfeld, A. M. (1965) Formal discussion of: Genetic factors in the etiology of cancer: An epidemiologic view. *Cancer Res.*, **25**, 1330.

Macklin, M. T. (1959) Comparison of the number of breast-cancer deaths observed in relatives of breast-cancer patients, and the number expected on the basis of mortality rates. *J. Natl Cancer Inst.*, **22**, 927.

Miettinen, O. S. (1972) Standardization of risk ratios. *Am. J. Epidemiol.*, **96**, 383.

Morgan, R. W., Vakil, D. V. & Chipman, M. L. (1974) Breast feeding, family history, and breast disease. *Am. J. Epidemiol.*, **99**, 117.

Papaioannou, A. N. (1974) *The Etiology of Human Breast Cancer: Endocrine, Genetic, Viral, Immunologic and other Considerations.* Berlin: Springer-Verlag. p. 47.

Penrose, L. S., Mackenzie, H. J. & Karn, M. N. (1948) A genetical study of human mammary cancer. *Br. J. Cancer*, **2**, 168.

Petrakis, N. L. (1977) Genetic factors in the etiology of breast cancer. *Cancer*, **39**, 2709.

Spiegel, E. (1918) Beitrage zur klinischen Konstitutionspatologie II. Organdisposition bei Ulcus pepticum. *Deutsches Arch. klin. Med.*, **126**, 45.

Thiessen, E. V. (1974) Concerning a familial association between breast cancer and both prostatic and uterine malignancies. *Cancer*, **34**, 1102.